# The Relationship Between the Need to Belong and Nature Relatedness: The Moderating Role of Independent Self-Construal

**DOI:** 10.3389/fpsyg.2021.638320

**Published:** 2021-02-11

**Authors:** Liman Man Wai Li, Mengru Liu, Kenichi Ito

**Affiliations:** ^1^Department of Psychology, The Education University of Hong Kong, New Territories, Hong Kong; ^2^Social Service Research Centre, Faculty of Arts and Social Science, National University of Singapore, Singapore, Singapore; ^3^School of Social Sciences, Nanyang Technological University, Singapore, Singapore

**Keywords:** nature relatedness, need to belong, self-construal, psychological needs, pro-environmental behavior

## Abstract

The perception of the relationship between humans and nature is important for promoting not only pro-environmental behaviors but also psychological well-being. The present research explored how people’s self-construal would moderate the relationship between the need to belong, the desire for social acceptance and connectedness and perceived nature relatedness. Two studies using community samples with diverse demographic characteristics in two different cultures (Study 1: the United States; Study 2: Singapore) obtained consistent findings. The results showed that independent self-construal, which emphasizes separateness from others in the social contexts, moderated the relationship between the need to belong and nature relatedness. Specifically, the need to belong was negatively associated with nature relatedness among people with a stronger independent self-construal, while this pattern was not significant among those with a weaker independent self-construal. No evidence for the moderating role of interdependent self-construal was found in the two studies. These findings highlighted the importance of non-nature experience in understanding people’s perception of human–nature relationships.

## The Moderating Role of Independent Self-Construal

Despite the severe environmental problems, people continue to engage in activities that damage the environment (Oskamp, 2000). The human–nature relationship becomes an important construct in environmental research because of its substantial influences on diverse pro-environmental values and behaviors (Schultz, 2000; Mayer and Frantz, 2004; Dutcher et al., 2007; Nisbet et al., 2009). The present research focused on nature relatedness, which refers to the way that individuals perceive, feel, and experience their connections with the natural environment (Mayer and Frantz, 2004; Nisbet et al., 2009). Previous work has put great efforts into identifying the antecedents of nature relatedness. For instance, the amount of time spent in nature (Schultz and Tabanico, 2007; Nisbet and Zelenski, 2011) and exposure to the natural environment (Kjellgren and Buhrkall, 2010; Valtchanov et al., 2010) predicted a higher level of nature relatedness.

While abundant evidence supports that nature experience is crucial for promoting nature relatedness (Brehm et al., 2006), its primary focus on nature-related external factors made the research on nature relatedness underdeveloped (Yang et al., 2018). In particular, research that explores whether experiences in non-nature domains, such as interpersonal experience and self-concept, can cause individual differences in nature relatedness is scarce. To fill this gap, the present research explored the relationship between people’s desire to be socially accepted by other people (i.e., need to belong; Baumeister and Leary, 1995) and nature relatedness and whether people’s self-construal would shape the examined relationship.

## The Relationship Between Need to Belong and Nature Relatedness

People differ in the level of need to belong (i.e., desire for social connectedness) (Leary et al., 2013). People are motivated to seek different means to satisfy their need to belong in social life because failing to meet this basic psychological need can lead to negative consequences on psychological well-being (Baumeister and Leary, 1995).

Fulfilling the need to belong can be achieved by gaining social acceptance, such as establishing new social relationships or maintaining stronger established social memberships (Williams, 2009). For instance, persons activated with a high need to belong showed greater conformity to others’ opinions (Williams et al., 2000), greater attention to information related to social connections (Pickett et al., 2004), and greater emphasis on social identities (Leary et al., 2013).

Some studies proposed that the desire for connectedness is not only found in the social domains. People also demonstrate a strong tendency to connect with non-humans, including pets (McConnell et al., 2011) and places (Scannell and Gifford, 2017). Following the biophilia hypothesis (Kellert and Wilson, 1993), humans have an innate desire to connect with nature, which is an important source of bonding and connection. Supporting this hypothesis, accumulative evidence demonstrates that nature relatedness is one important basic psychological need for humans (for a review, Baxter and Pelletier, 2019). Importantly, the desire for nature connectedness is independent of people’s belongingness obtained from social experiences (Brehm et al., 2006; Ryan et al., 2010; Zelenski and Nisbet, 2014). More interestingly, previous studies suggest that nature relatedness could be a potential compensatory mechanism for a strong desire for social connectedness, as a deprivation of one basic psychological need can be compensated by fulfilling other psychological basic needs (Ryan and Deci, 2017). In other words, it was likely that the need to belong (i.e., the desire for social connectedness) would be negatively associated with nature relatedness.

Although the relationship between the need to belong and nature relatedness has not been directly tested to the best of our knowledge, some studies provide evidence for a compensatory relationship between the need to belong and the desire for nature connectedness. People who experienced social exclusion showed greater intention for engaging in nature-related activities and a greater desire to connect with nature (Poon et al., 2015). More importantly, Yang et al. (2020) found that viewing nature scenes were effective in reducing the pain of social ostracism, which implicates that a stronger desire for social connection can be reduced by nature experience. These findings were consistent with the notion that deprivation of one basic psychological need, such as the desire for social connections, can be compensated by fulfilling other psychological basic needs, such as nature connectedness (Ryan and Deci, 2017).

## The Moderating Role of Independent Self-Construal

As discussed previously, people have a strong desire for belongingness, which can be reflected in different domains, including establishing emotional bonding with others (e.g., Leary et al., 2013) or with non-humans such as nature (e.g., Poon et al., 2015), and these means can be potentially compensatory to each other. However, which approaches are more likely to be adopted may vary across individuals. Previous research reveals that people’s response to various types of social experience depends on how they define themselves (e.g., Ren et al., 2013). For example, self-construal shapes how people interpret different social situations (Balcetis and Dunning, 2006). Therefore, we speculate that people’s self-construal may moderate the relationship between the need to belong (i.e., desire for social connectedness) and nature relatedness.

Self-construal reflects how people understand the relationship between themselves and other people in the surrounding environment. There are two major dimensions of self-construal: independent self-construal and interdependent self-construal. Although independent and interdependent self-construals are often discussed in the cross-cultural contexts, in which East-Asian cultures are likely to cultivate an interdependent self-construal while Western cultures are likely to cultivate an independent self-construal (e.g., Markus and Kitayama, 1991). The construct of self-construal is also meaningful at individual-level (e.g., Gabriel et al., 2007; He et al., 2012), as it reflects individual differences in the endorsement of independent self and interdependent self to capture the degree to which an individual is influenced by cultural norm, values, and beliefs in his/her self-concept (Singelis, 1994). In other words, it can capture the individual differences in the endorsement of the shared beliefs or intersubjective knowledge of a given culture (Chiu et al., 2010). Existing evidence shows that the influence of these two dimensions of self-construal is independent of one another (Ren et al., 2013; Ho and Kong, 2016). An independent self-construal emphasizes autonomy and separateness from others (Markus and Kitayama, 1991; Cross et al., 2011); thus, the desire of connecting with others may not be a primary goal to people with a stronger independent self-construal (Loveland et al., 2010). As a result, the nature relatedness would be adopted as a compensatory mechanism for the desire of social connectedness among people with a stronger independent self-construal than those with a weaker independent self-construal.

Some work provides support for the above theorizing. The level of satisfaction during shopping experienced among highly independent people was found to be less dependent on the presence of others (He et al., 2012). Similarly, reminding close relationships did not bring significant benefits to people activated with an independent self-construal (or even brought a marginally significant negative impact to those people) (Gabriel et al., 2007). Because of the less primary interpersonal goal shared among highly independent people, it is likely that they would be more motivated to adopt a non-interpersonal way to achieve their sense of connectedness, e.g., through nature relatedness. This would result in a stronger negative correlation between the desire for social connectedness and nature relatedness among more independent people.

In contrast to independent self-construal, interdependent self-construal emphasizes connectedness with other people (Markus and Kitayama, 1991; Cross et al., 2011). Among people with a strong interdependent self-construal, establishing emotional bonding with non-humans may not be important. On the one hand, they are highly motivated to establish emotional bonding with different persons or groups (White et al., 2012). Therefore, they were found to have more close relationships with others (Yeung et al., 2008). On the other hand, the salience of close interpersonal relationships was found to bring significant benefits to those with an interdependent self-construal (Gabriel et al., 2007). Because of the available support from close others among interdependent people, connecting with non-humans as a strategy to meet the need to belong may not be highly relevant. Therefore, we did not make any specific hypotheses on the moderating role of interdependent self-construal.

## The Current Research

To summarize, we explored whether independent self-construal would moderate the relationship between the need to belong and nature relatedness. As the need to belong is fundamental to humans (Baumeister and Leary, 1995), people with strong independent self-construal, who emphasize separateness and autonomy from others in the social contexts (Markus and Kitayama, 1991), may be more likely to build their sense of belongingness through connecting themselves with nature, i.e., a non-interpersonal way. Therefore, we expected that highly independent people would demonstrate a stronger negative association between the need to belong and nature relatedness.

To test our hypotheses, we first conducted Study 1 through Amazon Mechanical Turk (MTurk) in the United States. To ensure the generalizability of the obtained findings in different societies and populations with diverse demographic characteristics, we conducted Study 2 in another culture, i.e., Singapore, with a larger community sample.

## Study1

### Method

#### Participants

With an expected small-to-medium interaction effect (*f* = 0.20), we need to have about 200 participants to reach 80% power using G^∗^Power (Faul et al., 2009). We set 200 participants as the target sample size. To recruit participants with diverse demographic information, we collected data via MTurk in the United States. Finally, 204 participants (87 males, 117 females; *M* = 36.58, SD = 12.72, range: 21–76) completed the online survey. Informed consent was obtained from all individual participants included in the study. Among the participants, 76% of participants were of European descent, 9% were of African descent, and 5% were of Hispanic descent. The majority of participants were currently full-time (65.2%) or part-time (16.7%) employees and had obtained degrees from post-secondary institutions (69.1%).

#### Scales

Participants responded to online questionnaires that measure their need to belong, self-construal, and nature relatedness. The questionnaires were randomly ordered. The questionnaires and methodology for this study was approved by the Human Research Ethics committee of the last author’s university (IRB 2019-12-032).

##### Need to belong

We adapted ten items from Leary et al. (2013) to measure respondents’ needs to connect with others and belong to a group. The sample items include, “I try hard not to do things that will make other people avoid or reject me” and “If other people don’t seem to accept me, I don’t let it bother me.” (a reverse-scored item). Participants indicated their agreement with each statement on a scale from 1 (strongly disagree) to 5 (strongly agree). The reliability was satisfactory (α = 0.80). The scores of all items were averaged to form a need to belong score, with a higher score indicating a higher level of need to belong.

##### Nature relatedness

We adapted 21 items from Nisbet et al. (2009) to measure how strong respondents are connected with the natural world. The sample items include, “I am not separate from nature, but a part of nature” and “I don’t often go out in nature” (a reverse-scored item). Participants indicated their agreement with each statement on a scale from 1 (strongly disagree) to 5 (strongly agree). The reliability was satisfactory (α = 0.87). The scores of the items were averaged to form a nature relatedness score, with a higher score indicating stronger nature relatedness.

##### Self-construal

We adopted the self-construal measure developed by Singelis (1994). Although we primarily focused on the moderating role of independent self-construal, we included all 24 items in the study. Independent self-construal was measured by 12 items (α = 0.78). A sample item includes, “I act the same way no matter who I am with.” Interdependent self-construal was measured by 12 items (α = 0.82). A sample item includes, “My happiness depends on the happiness of those around me.” Participants indicated their agreement with each statement on a scale from 1 (*strongly disagree*) to 7 (*strongly agree*). Separate scores were computed for independent self-construal and interdependent self-construal with a higher score indicating a higher level in that dimension.

### Results

Table 1 summarizes the descriptive statistics, including means, standard deviations, and zero-order correlation coefficients for all measures used in Study 1^1^.

**TABLE 1:** Means, standard deviation (SD), and intercorrelations for all measures in Studies 1 and 2.

	Mean (SD)	1	2	3	4
**Study 1**					
1. Need to Belong	3.12 (0.69)	–			
2. Nature Relatedness	3.52 (0.64)	–0.07	–		
3. Independent self-construal	5.25 (0.79)	–0.12	0.15*	–	
4. Interdependent self-construal	4.93 (0.87)	0.42***	0.08	0.35***	–
**Study 2**					
1. Need to Belong	3.15 (0.58)	–			
2. Nature Relatedness	3.20 (0.29)	−0.08*	–		
3. Independent self-construal	4.92 (0.72)	–0.04	0.10**	–	
4. Interdependent self-construal	4.93 (0.69)	0.30***	0.14***	0.42***	–

*p < 0.05; ***p < 0.001.

A regression analysis was conducted to explore the moderating role of self-construal. The major predictors with continuous scores were centered at their own mean before computing associated interaction terms. The scores of the need to belong, independent self-construal, interdependent self-construal, the interaction of the need to belong and independent self-construal, and the interaction of the need to belong and interdependent self-construal were entered into the regression model. Previous work demonstrated that demographic variables are important predictors of pro-environmental related outcomes (Gifford and Nilsson, 2014). To minimize the potential confounding effect of demographic variables, we controlled for the effect of participants’ gender, age, and education level in the analysis. Table 2 summarizes the results of the analysis. Refer to Table 2 for the effect of control variables.

**TABLE 2:** The results of regression analyses in Studies 1 and 2.

	Study 1		Study 2	
	*b* (SE)	β	*b* (SE)	β
Age	0.007 (0.004) ^†^	0.13	−0.001 (0.001)	−0.03
Gender	0.16 (0.09) ^†^	0.13	0.04*(0.02)	0.08
Educational level	0.001 (0.02)	−0.001	0.01* (0.01)	0.06
Need to belong	−0.12 (0.08)	−0.12	−0.07***(0.02)	−0.14
Independent self-construal	0.09 (0.06)	0.12	0.01 (0.01)	0.02
Need to belong × independent self-construal	−0.22**(0.07)	−0.22	−0.07***(0.02)	−0.13
Interdependent self-construal	0.07 (0.06)	0.11	0.08*** (0.02)	0.19
Need to belong × interdependent self-construal	−0.07 (0.07)	−0.07	−0.01 (0.02)	−0.01
*R*^2^	0.13***		0.06***	

Unstandardized coefficients (*b*), standard error (SE), and standardized coefficients (β) are reported. ^†^*p* = 0.06; *p < 0.05; **p < 0.01; ***p < 0.001.

**FIGURE 1:**
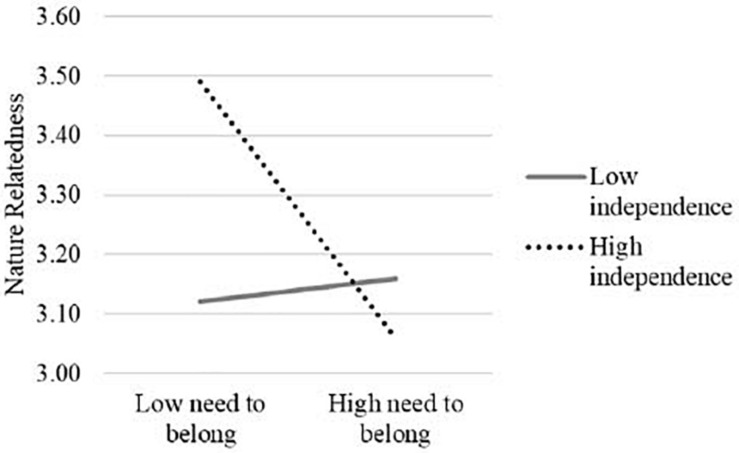
The interaction effect of the need to belong and independence in predicting nature relatedness in Study 1.

The results showed that the score of need to belong was non-significant but negatively associated with nature relatedness, *b* = −0.12, SE = 0.08, *p* = 0.14. The main effect of independent self-construal was not significant in predicting nature relatedness, *b* = 0.09, SE = 0.06, *p* = 0.13. Importantly, the interaction of the need to belong and independent self-construal was significant, *b* = −0.22, SE = 0.07, *p* = 0.002. The simple slope analyses showed that the need to belong was negatively associated with nature relatedness among individuals with a stronger independent self-construal (1SD above the mean), *b* = −0.29, SE = 0.10, *p* = 0.004, whereas the association between the need to belong and nature relatedness was not significant among participants with a weaker independent self-construal (1SD below the mean), *b* = 0.06, SE = 0.10, *p* = 0.52 (see Figure 1). In contrast, the main effect of interdependent self-construal, *b* = 0.08, SE = 0.06, *p* = 0.205, and the interaction of the need to belong and interdependent self-construal, *b* = −0.07, SE = 0.07, *p* = 0.344, were not significant.

### Discussion

Study 1 provided evidence to support the moderating role of independent self-construal in shaping the relationship between the need to belong and nature relatedness. Specifically, a significant negative association between the need to belong and nature relatedness was observed only among people with a stronger independent self-construal but not among those with a weaker independent self-construal. In addition, there was no evidence for the moderating role of interdependent self-construal.

Despite the supportive evidence in Study 1, the demographic characteristics of the participants were not as diverse as we expected. For instance, all participants were above 20 years old. In addition, it was unknown how generalizable the results can be in different societies, where promote different cultural values and societal norms. To replicate the findings in Study 1, we conducted Study 2 in Singapore with a community sample.

## Study2

### Methods

#### Participants

As a part of a large study, a total of 959 participants (483 male participants, 476 female participants) were recruited through a marketing company in Singapore. Participants’ age ranged from 13 to 65 years old (*M* = 35.50, SD = 11.73). A majority of participants were employees (full-time: 71.8%; part-time: 8.9%), and 11.1% of the participants were students. Informed consent was obtained from individual participants or their legal guardians if participants were below 18 years old.

Participants completed a set of questionnaires, including Need to Belong (Leary et al., 2013; α = 0.79), Nature Relatedness (Nisbet et al., 2009; α = 0.72) (see text footnote 1), and Independent-Interdependent Self-Construal (Singelis, 1994; Independence: α = 0.79; Interdependence: α = 0.81). These scales were identical to Study 1. They also completed other scales related to pro-environmental tendencies, which were used in other studies. The questionnaires and methodology for this study was approved by the Human Research Ethics committee of the last author’s university (IRB 2017-05-032-01).

### Results

Table 1 summarizes the descriptive statistics of all measures in Study 2^2^. We followed the same analytic procedure used in Study 1 to test the moderating role of self-construal. Table 2 summarizes the results of the regression analyses. Refer to Table 2 for the effect of control variables (i.e., age, gender, and educational level).

**FIGURE 2:**
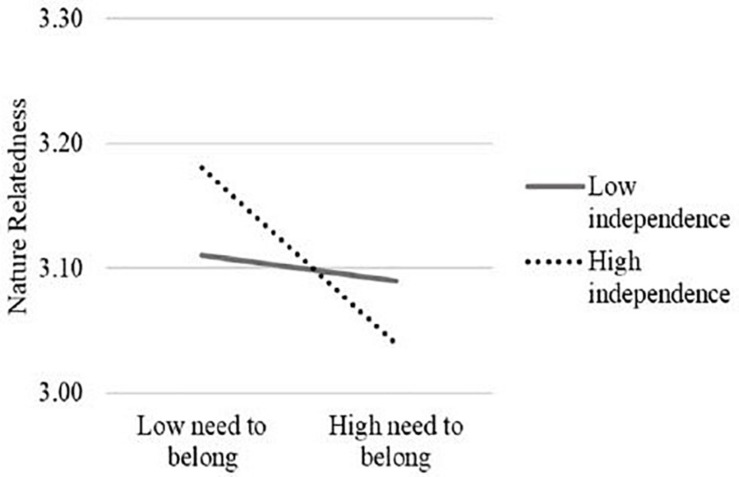
The interaction effect of the need to belong and independence in predicting nature relatedness in Study 2.

The results showed that the need to belong was negatively associated with nature relatedness, *b* = −0.07, SE = 0.02, *p* < 0.001. The main effect of independent self-construal was not significant in predicting nature relatedness, *b* = 0.01, SE = 0.01, *p* = 0.678. Importantly, the interaction of the need to belong and independent self-construal was significant, *b* = −0.07, SE = 0.02, *p* < 0.001. The simple slope analyses showed that the score of need to belong was negatively associated with nature relatedness among individuals with a stronger independent self-construal (1SD above the mean), *b* = −0.12, SE = 0.02, *p* < 0.001, whereas the association between the need to belong and nature relatedness was not significant among participants with a weaker independent self-construal (1SD below the mean), *b* = −0.02, SE = 0.02, *p* = 0.506 (see Figure 2).

In contrast, the main effect of interdependent self-construal was significant, *b* = 0.08, SE = 0.02, *p* < 0.001. However, the interaction of the need to belong and interdependent self-construal was not significant, *b* = −0.01, SE = 0.02, *p* = 0.694.

### Discussion

With the data from a community sample in Singapore, Study 2 replicated the patterns obtained in Study 1. Specifically, a significant negative association between the need to belong and nature relatedness was observed among people with a stronger independent self-construal but not among those with a weaker independent self-construal. Similar to Study 1, we did not observe any evidence suggesting the moderating role of interdependent self-construal for the relationship between the need to belong and nature relatedness.

## General Discussion

The present research explored whether nature relatedness can be a compensatory mechanism for the need to belong, especially among highly independent people. The results in Studies 1 and 2 using different cultural samples provided supportive evidence. Among people with a strong independent self-construal, a lower level of need to belong was significantly associated with a higher level of nature relatedness. In contrast, this pattern was not significant among people with a weak independent self-construal. The stronger negative association between need to belong and nature relatedness among people with a strong independent self-construal may indirectly imply that nature relatedness could be used as a compensatory mean for the need to belong (i.e., the desire for social connectedness).

This research integrates the theories and findings of social psychology and environmental research. On the one hand, people are motivated to seek fulfilling other basic psychological needs due to the deprivation of one specific need (Ryan and Deci, 2017). Therefore, as one of the basic psychological needs (Baxter and Pelletier, 2019), previous work demonstrates that nature relatedness can be compensatory for the need to belong (e.g., Poon et al., 2015). Extending previous work, the present research suggests that this strategy may be more likely to be adopted by people with a strong independent self-construal, who value separateness from others and individuality (Markus and Kitayama, 1991; Cross et al., 2011).

## Implications

The present research has some implications for environmental studies. Similar to the research on pro-environmental behavior (e.g., Tam, 2013; Eom et al., 2016), research on nature relatedness mostly focused on exploring nature-related antecedents. Recent studies (e.g., Leung et al., 2015; Chuang et al., 2016; Ito and Li, 2019; Ito et al., 2020; Li et al., 2020) provided supportive evidence for the importance of domain-general factors in shaping people’s response to different environmental issues. To further evaluate the importance of domain-general factors in environmental research, the present research explored the moderating role of self-construal on the relationship of need to belong and nature relatedness. Some evidence supports that self-concept can directly affect the degree of pro-environmental tendency (e.g., Chuang et al., 2016). Extending previous studies, we found some evidence that independent self-construal can also exert a moderating effect in shaping the relationship of a factor that stresses interpersonal attachment (i.e., the need to belong) with nature relatedness. These findings highlight the complexity of the influence of domain-general factors on shaping people’s responses to environmental issues.

The present research provides further evidence to demonstrate the importance of nature relatedness. Previous work showed that nature relatedness predicted better psychological well-being, including greater positive affect (e.g., Capaldi et al., 2014), better stress recovery (e.g., Valtchanov et al., 2010), and a stronger feeling of personal growth (e.g., Zelenski and Nisbet, 2014). The present research found a negative association between the need to belong and nature relatedness (though it was only observed among highly independent people), which suggests that nature relatedness may be one effective compensatory mechanism to satisfy the need to belong. These findings were additional evidence for a recent proposal proposing that nature relatedness is a basic psychological need (Baxter and Pelletier, 2019), which highlights the innate tendency of connecting to nature among humans (Kellert and Wilson, 1993).

Finally, the present research may provide some practical implications. Previous studies showed that social rejection increased people’s desire to connect with nature (e.g., Poon et al., 2015). The present research found a negative association between the need to belong and nature relatedness, especially among highly independent people that perceive separation from others (Markus and Kitayama, 1991). Taken together, it suggests that nature is more beneficial to those who find it challenging to establish emotional bonding in social interactions. For instance, connecting with nature, which helps build emotional bonding (Kellert and Wilson, 1993), could be an effective way to promote a sense of belongingness, which could, in turn, promote better psychological well-being among people with high social anxiety that are likely to perceive a lower level of social support (Indian and Grieve, 2014). In other words, it is possible that nature experience would be very effective in promoting psychological well-being among people who are vulnerable to social challenges. Further studies should explore this possibility.

## Limitations and Future Directions

Some limitations required further considerations. First, no causal claims could be made based on the correlational data. Experimental studies are needed for causally testing whether independent people are indeed more likely to use nature relatedness as a compensatory mechanism for the need to belong. Second, although a consistent pattern of results was obtained between the two studies, we used identical measures. Future studies should adopt different measures, such as behavioral measures or less explicit measures, to further evaluate the replicability of the obtained findings. Third, following the theories related to basic psychological needs to humans (Ryan and Deci, 2017), there are other types of basic psychological needs such as autonomy. To comprehensively examine the role of nature relatedness as one fundamental psychological need, future studies should examine whether nature relatedness would be useful to compensate for the deprivation of other basic psychological needs.

## Data Availability Statement

The raw data supporting the conclusions of this article will be made available by the authors, without undue reservation.

## Ethics Statement

The studies involving human participants were reviewed and approved by Human Research Ethics Committee Nanyang Technological University, Study 1: IRB 2019-12-032 Study 2: IRB 2017-05-032-01. Written informed consent to participate in this study was provided by the participants’ legal guardian/next of kin.

## Author Contributions

KI conceived of the original idea with the help of LMWL, KI, and ML carried out the study. KI, LMWL, and ML analyzed the data. KI and LMWL wrote the manuscript. All authors listed have made a substantial, direct and intellectual contribution to the work, and approved it for publication.

## Conflict of Interest

The authors declare that the research was conducted in the absence of any commercial or financial relationships that could be construed as a potential conflict of interest.

## Footnotes

1 Unexpectedly, we found a significant positive correlation between independent and interdependent self-construal, *r*(202) = 0.35, *p* < 0.001 in Study 1 and *r*(957) = 0.35. *p* < 0.001 in Study 2. A positive correlation between the independent and interdependent self-construal was observed in the past study with different cultural groups (e.g., Fernández et al., 2005). Besides, positive self-view was theorized to be associated with the positive evaluation of interpersonal relationships elsewhere (Leary, 2005). Thus, the results were consistent with past studies.

2 We removed 14 items because the total item correlation was below 0.2 (Everitt, 2002). The remaining seven items were averaged to form a nature relatedness score with a higher score indicating stronger nature relatedness. Excluded items:

**Table:**

1.	I enjoy being outdoors, even in unpleasant weather.
2.	My ideal vacation spot would be a remote, wilderness area.
3.	I always think about how my actions affect the environment.
4.	I enjoy digging in the earth and getting dirt on my hands.
5.	My connection to nature and the environment is a part of my spirituality.
6.	I am very aware of environmental issues.
7.	I take notice of wildlife wherever I am.
8.	I don’t often go out in nature.
9.	The thought of being deep in the woods, away from civilization, is frightening.
10.	Even in the middle of the city, I notice nature around me.
11.	My relationship to nature is an important part of who I am.
12.	The state of non-human species is an indicator of the future for humans.
13.	I think a lot about the suffering of animals.
14.	I feel very connected to all living things and the earth.
